# Half-Hour Recreations in Popular Science

**Published:** 1872-08

**Authors:** 


*Half-Hour Recreations in Popular Science*, Nos. 3 and 4, DANA ESTES, Editor. Boston: LEE & SHEPARD, 1872.

Number Three of these popular essays on Science is devoted to an explanation of spectrum analysis, including also the theory of sound, heat, light and color; taken from the writings of Schellen, Roscoe, and Huggins. The explanations which are here afforded, are finely adapted to the popular mind and are so entirely free from technical terms and expressions, that they can be readily understood by all, except the most careless and superficial reader.

Number Four contains an account of some of the discoveries which have been made through the agency of spectrum analysis, and is also made up in large part from the works of Schellen, Roscoe, Young, and others.

The illustrations which are introduced into each volume are finely conceived and ably executed, and add much to the general worth of the books. Messrs. Lee & Shepard are publishing a series of these books under the editorial management of Mr. Estes, and they will in our opinion go far to meet the public demand for popular essays on scientific subjects. If the succeeding numbers are as interesting as the two before us, they will without doubt meet with a cordial welcome. We bespeak for both the editor and publishers the support of the public in their endeavor to popularize scientific subjects.

